# Case Study of Sequence Capture Enrichment Technology: Identification of Variation Underpinning Developmental Syndromes in an Amniote Model

**DOI:** 10.3390/genes3020233

**Published:** 2012-03-26

**Authors:** Elizabeth A. Robb, Mary E. Delany

**Affiliations:** Department of Animal Science, University of California, Davis, CA 95616, USA; E-Mail: earobb@ucdavis.edu

**Keywords:** capture array, next generation sequencing, bioinformatics, chicken, mutation, SNP, variant, indel, congenic

## Abstract

Chicken developmental mutants are valuable for discovering sequences and pathways controlling amniote development. Herein we applied the advanced technologies of targeted sequence genomic capture enrichment and next-generation sequencing to discover the causative element for three inherited mutations affecting craniofacial, limb and/or organ development. Since the mutations (*coloboma*, *diplopodia-1* and *wingless-2*) were bred into a congenic line series and previously mapped to different chromosomes, each targeted mutant causative region could be compared to that of the other two congenic partners, thereby providing internal controls on a single array. Of the ~73 million 50-bp sequence reads, ~76% were specific to the enriched targeted regions with an average target coverage of 132-fold. Analysis of the three targeted regions (2.06 Mb combined) identified line-specific single nucleotide polymorphism (SNPs) and micro (1–3 nt) indels. Sequence content for regions indicated as gaps in the reference genome was generated, thus contributing to its refinement. Additionally, Mauve alignments were constructed and indicated putative chromosomal rearrangements. This is the first report of targeted capture array technology in an avian species, the chicken, an important vertebrate model; the work highlights the utility of employing advanced technologies in an organism with only a “draft stage” reference genome sequence.

## 1. Introduction

Next-generation sequencing (NGS) enables researchers to accurately and rapidly address many critical and outstanding biological questions regarding the relationship between genotype and phenotype. Employment of a targeted genomic enrichment capture array (CA) approach can advance such research contingent on sequence knowledge of the genome of interest or a closely related genome. Paired with comparative developmental biology, NGS can elucidate the sequences responsible for vertebrate developmental malformations [1]. Like many laboratories worldwide, we were interested in employing the latest technologies, in a cost-effective manner, to understand a biological system despite lacking substantial resources or expertise in bioinformatics. Our genome of interest, the chicken, has not been mapped to the precision, detail, or accuracy of that of the mouse or human [2,3,4]. To this end, we employed a commercial service provider and made optimal use of specialized genetic resources to identify sequence variation associated with mutations for the long-term objective of determining the causative elements and their role in amniote developmental pathways. 

The University of California-Davis (UCD) maintains a series of developmental mutant chicken lines well-studied for phenotype and mode of inheritance [1,5,6,7]. The mutations are single-gene recessives causing craniofacial, limb, skeletal, muscular and/or integument abnormalities having homology with developmental syndromes in human. The mutations were bred on the same inbred genetic background (UCD-003) to generate congenic (a.k.a. coisogenic) inbred lines, thereby providing an advantage for discovery of the specific genetic element causing each defect. The chromosomal locations and causative regions (CR; a.k.a. linked region) associated with each of the three mutations were previously mapped using SNP genotyping arrays (*diplopodia-1* (*dp-1*) mapped to GGA 1; *wingless-2* (*wg-2*) mapped to GGA 12; *coloboma* (*co*) mapped to GGA Z, sex-linked) [1,8].

**Figure 1:**
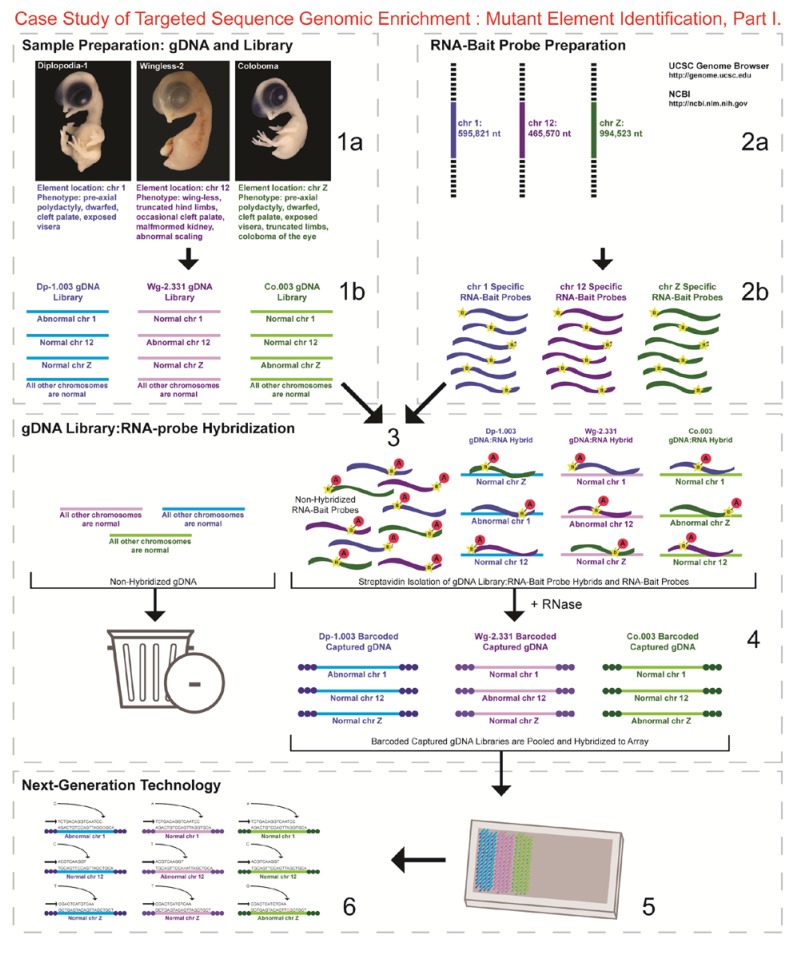
Targeted Sequence Genomic Enrichment Methodology, Part I. Targeted genomic capture enrichment paired with next-generation sequencing technology was utilized to sequence, in their entirety, the three chromosomal regions associated with three developmental mutations in the chicken. Approximate timeline from contract with the service provider to transfer of data (steps 1–6) was ~6 months.

Here, we utilized capture enrichment technology rather than exome or whole genome sequencing (WGS) for several reasons, including: (1) variability in the phenotypic expression of each syndrome suggested that the causative element could reside within a regulatory element rather than exon; (2) the reference chicken genome sequence is still in an early stage (gene annotation not as robust) thereby possibly leading to missed genes; (3) we have the advantage of congenic lines and knowledge of specific regions associated with each mutation, therein WGS was unnecessary; and (4) at the time, the cost for WGS three mutant lines was higher compared to the capture array technology. Therefore, we targeted the specific genomic coordinates of the three CRs on GGA 1, 12, and Z, totaling 2.06 Mb, to sequence, in their entirety, those regions known to maintain the elements causing the three unique developmental mutations. The CA technology consists of a “targeted-genomic” aspect wherein overlapping oligonucleotide RNA-bait probes are generated for a specific genomic segment (in our case, maximum CR linked to each mutation). The probes are hybridized to DNA from the target (in our case, three developmental mutants barcoded for line-specificity), amplified and sequenced using NGS methods in the “capture enrichment” aspect of the technology (Figure 1 and Figure 2). The bioinformatics analyses identified genetic differences (e.g., SNPs, micro-indels, and macro-indels) providing information on both normal variation from the introgressed region(s), as well as priority mutant-specific sequences for future functional studies.

1a. *Identified regions for targeted sequence capture enrichment.* The linked/causative regions were previously identified using SNP arrays [1]. High quality, high molecular weight genomic (g) DNA was isolated from specific samples of interest (Agilent SureSelect kit suggestions: 10–20 μg of purified, non-amplified gDNA per sample with concentrations between 100–1,000 ng/μL, A_260_/A_280_ ratio >1.8). The samples were assessed for quality (e.g., spectrophotometric analysis, agarose gel quality control). The quality of the gDNA and library (e.g., library size distribution and concentration) generated will have an impact on the quality of the sequence results.
1b. *Prepared genomic libraries.* Three individual libraries were generated from gDNA isolated from three developmental mutant congenic lines using Agilent’s SureSelect Target Enrichment System. The following steps were taken in the preparation of each individual mutant library: (i) shear DNA to obtain fragments with a base pair peak of 150 to 200; (ii) blunt-end fragments with 5′-phosphorylated ends; (iii) attach a dATP to the 3′ end of the DNA fragments. After dATP nucleotides are added to the 3′ end of the DNA fragments; (iv) adaptors (specific to the sequencing platform) are ligated to the 3′ dATP overhang; (v) a library pre-enrichment amplification followed by (vi) a library quality control and quantitation assessment with a Bioanalyzer and PicoGreen assay. Please note that a purification procedure occurs in between each of the library preparation steps (i–vi). If the initial or enriched template library contains low amounts of nucleic acid, one can amplify the library before sequencing using PCR and a polymerase that is not biased as to template size. One can outsource any of the subsequent steps or perform them in the research laboratory.
2a. *Designed overlapping RNA-bait probes (120 nt in length) for sequencing for the region of interest.* Sequence information for chromosomes 1 and 12 was obtained from NCBI (WASHUC2, May 2006 [2]) while red jungle fowl, UCD-001 (reference genome genetic line), sequence data for chromosome Z was obtained from Dr. D. Winston Bellott and Dr. David Page prior to NCBI submission [9]. We provided SeqWright Inc., with coordinate information or sequence content to design and create the RNA-bait probes that complemented the three targeted chromosomal regions of interest.
2b. *Generated overlapping biotinylated RNA-bait probes specific to each region of interest.* RNA library “baits” were generated for bead capture purposes (step 3).
3. *Hybridized denatured gDNA library fragments (150–200 nt) to RNA-bait probes (120 nt).* RNA-baits were hybridized to gDNA in order to enrich for complimentary DNA sequence information specific to the three regions of interest. Streptavidin coated magnetic beads were utilized to capture RNA-bait:gDNA-library fragment hybrids as a means to separate those DNA fragments not complementary to the targeted regions. Beads were washed and digested (RNased) to isolate only gDNA library fragments that hybridized to RNA-bait probes.
4. *Barcoded samples (pooled or individual).* In order to identify each individual or group, samples were barcoded (a.k.a. index-tagged). In the case of this project, Co.003 gDNA (2 pooled female mutants), Dp-1.003 (2 pooled female mutants), and Wg-2.331 (2 pooled female mutants), each had an individual barcode unique to the genetic line. [Prior to sequence read alignment and bioinformatic analysis, the barcoded sequencing reads were first sorted and the barcode was then removed.]
5. *Pooled barcoded samples, hybridized sequences to array, and amplified DNA prior to sequencing.* All three genetic lines were pooled into one sample with each barcoded sample present in equimolar amounts. Pooled, barcoded libraries (single-stranded) were hybridized to an array (in the case of this project, we used one-fourth of a slide) utilizing the adaptors (see step iv in 1b) previously incorporated at the end of the DNA sequence. Unlabeled nucleotides and enzyme were added to initiate solid-phase bridge amplification (this generates double-stranded bridge molecules), DNA was then denatured, and amplification to generate sequence clusters proceeded.
6. *Next-generation sequencing.* Labeled dNTP reversible terminators (one base at a time), primers and DNA polymerase were added to the slide and sequenced using laser excitation. This step was repeated until each barcoded DNA fragment was sequenced. For this project, SOLiD™ version 3-Plus using 50 ligation cycles (50 base pair sequencing) was employed. A total of 3.64 Gbp of sequence data was generated.

**Figure 2:**
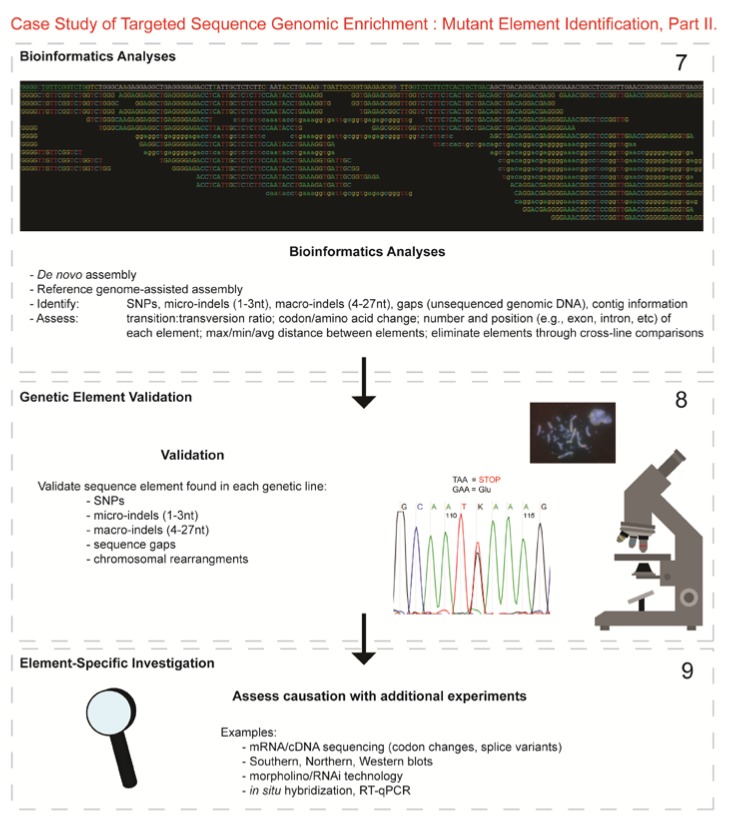
Targeted Sequence Genomic Enrichment Methodology, Part II. Analysis of the targeted genomic capture enrichment and next-generation sequencing data allowed for the identification of variants and chromosomal rearrangements which were further validated using new mutant samples in order to identify the causative element for each of the developmental mutations. Approximate timeline from obtaining the raw SOLiD™ (colorspace) sequence reads to validating the sequence variants identified (steps 7–8) was 6 months.
7. *Bioinformatics.* We received colorspace reads and quality value files (both in FASTA-like formats) from SeqWright, Inc. SNPs, micro-indels (1–3 nt), macro-indels (4–27 nt) and gaps were identified for each of the three genetic lines. Several reference-assisted *de novo* assemblies were generated using Mauve 2.3.1c software [10] in order to identify chromosomal rearrangements. Additional variant analyses included, but were not limited to, identification of: transition:transversion ratios, codon and amino acid modifications due to presence of the element under study, number of each element and maximum/minimum/average distance between variants, as well as position within the genome (e.g., exon, intron, splice site, *etc*.).
8. *Validated genetic elements*. Variations were assessed for linkage to the mutant and to evaluate the contribution of the polymorphisms to the mutant phenotype and/or genetic line using a new cohort of individuals (n ≥ 20) [11].
9. *Element-specific investigation.* Each genetic element must be further assessed to discover causation towards the phenotype. Examples of additional assays include: mRNA/cDNA sequencing (codon/amino acid modifications, splice variants), Southern, northern, western blots, morpholino/RNAi, *in situ* hybridization, RT-qPCR, and/or chromatin studies.

## 2. Results and Discussion

The development of NGS technologies is advantageous for all genomics research, especially for those genomes not as well annotated as mouse and human. Such technologies are of interest to laboratories of varied size, scope, and funding resources. NGS is no longer limited to novel sequence identification, but also offers insight into sequence variation, quantitative gene expression analysis, and questions of evolutionary genomics. Applications of the CA technology include but are not limited to re-sequencing of exonic regions, candidate gene sets, and large genomic loci as well as biomarker discovery and genetic marker development. Further, CA can provide a better understanding of the genetic basis for polygenic diseases, metabolic pathways, and in the case of this study, sequencing of unique genetic lines. To date, CA studies have been reported in mouse and human for the aforementioned applications, as well as a few domesticated species with many more plant and animal systems in the pipeline [12,13,14,15,16,17,18,19,20,21]. CA studies have led to the discovery of more than 40 Mendelian disorders in human [22].

Here, we describe the utilization of targeted sequence genomic enrichment in an important vertebrate model with a relatively early-stage reference genome [2], taking advantage of congenic inbred lines. Our long-term objective is to utilize the information gained to establish the specific element or gene responsible for each of the inherited recessive mutations known to result in abnormal developmental phenotypes [1]. This technology was successful in identifying numerous sequence elements unique to each genetic line in addition to reducing the size of the linked region (Table 1 and Table 2), thereby eliminating some candidate genes. Figure 1 and Figure 2 present a schematic, displaying the methodology utilized in our study, and provides a step-wise path for other researchers. Figure 1 begins with the collection and preparation of samples and the genomic libraries (step 1a, b), describes the design and generation of RNA-bait probes (step 2a, b) and the subsequent hybridization of the probes to the individual libraries (step 3) for genomic enrichment. The enriched libraries are then index-tagged (for identification purposes) (step 4) and sequenced (step 5, 6). Figure 2 describes the bioinformatics analyses conducted (step 7) and discusses additional functional assays (steps 8, 9).

**Table 1:** Statistics of the three regions captured using the targeted genomic capture enrichment technology.

Genetic Lines	Total Sequenced Region					
	Chr	Size (nt)	No. Genes	No. Gaps ^A^	NCBI Mean Quality Score	GC richness
Diplopodia-1.003	1	595,821	19	5	87.3%	39.6%
Wingless-2.331	12	465,570	13	15	94.5%	41.7%
Coloboma.003	Z	994,523	6	0	94.5%	39.4%
Total	-	2,055,914	38	20	-	-
Average	-	685,305	12.7	7	92.1%	40.2%

Three unique chromosomal regions were targeted for utilization in the genomic capture enrichment technology. Descriptive measures include targeted chromosome and size, number of genes and sequence gaps found within targeted region, percent GC richness and quality score. ^A^ Gaps were identified through assessment of each linked, genomic region for the three mutations using the UCSC Genome Browser [4]. Gaps identified for the Dp-1.003 chromosome 1 region were ≤1000 nt while Wg-2.331 region gaps were ≤1500 nt. No gaps were present in the 995 kb chromosome Z region for the Co.003 genetic line. However, given the repetitive nature of the Z chromosome, probes were only generated for 990,270 of the 994,523 nts. Note that the 4253 nts were present in a genomic region shown to no longer be linked to the *Co*.003 mutation [1].

### 2.1. Three Region (2.06 Mb) Sequencing and Mapping

SeqWright’s Genomic Enrichment Services were employed to design a custom NimbleGen capture array to enrich for sequencing the chromosomal regions linked to three developmental mutations in the chicken. Using only one-fourth of a slide and SOLiD ™ version 3-Plus using 50 ligation cycles (50 bp sequencing) a total of 3.64 Gbp of sequence data were generated. Using a short read mapping program, BWA, 72.6% of all reads were mapped to the three linked chromosomal segments; these mapped reads covered 1.95 Mb of the combined 2.06 Mb probe-generated regions (Table 2).

### 2.2. Analysis of Three Congenic Developmental Mutant Genomes

The following elements were assessed from the comparisons among the three genetic mutant lines using several programs and custom scripts (see Methods): SNPs, insertions (1–3 nt), deletions (1–3 nt), and gaps (unsequenced regions). A total of 2593, 1724, and 2500 SNPs were found within the three mutant CRs (GGA 1, 12, and Z) for dp-1.003, wg-2.331, and co.003, respectively. The insertions and deletions were 150 and 133, respectively, for dp-1.003 on GGA 1, 108 insertions and 138 deletions for wg-2.331 (GGA 12), and for co.003, a total of 125 insertions and 155 deletions were identify within the CR on GGA Z (Table 3). Only those variants localized within the 2.06 Mb targeted region are reported, all others are considered non-specific.

**Table 2:** Summary of targeted genomic enrichment sequencing results for three developmental mutant congenic lines.

Genetic Lines	Sequencing Read Statistics					Region Reduction (Post-Analysis)		
	Total Reads Generated ^A^	Total Mapped Reads ^B^	Average Coverage ^C^	Region Sequenced ^D^	No. Coverage Gaps ^E^	Remaining Size (nt)	Fold Reduction	No. Genes Remaining
Diplopodia-1.003	21.0M	15.5M	107.2×	96.9%	232	261,947	2.3×	12
Wingless-2.331	36.0M	28.3M	217.1×	85.3%	274	259,545	1.8×	13
Coloboma.003	15.7M	11.9M	72.1×	98.4%	525	306,847	1.3×	5
Total	72.7M	55.7M	-	-	1,031	828,339	-	30
Average	24.2M	18.6M	132.1×	93.5%	344	276,113	1.8×	10

Targeted genomic capture enrichment results: read statistics and post-analysis assessment. Descriptive measures include: total reads generated, total reads mapped to targeted region (2.06 Mb), average fold coverage, percentage region sequenced, and number of sequence/coverage gaps. Assessment of the sequence reads resulted in a reduction in the linked-region size. ^A^ Total number of reads generated, M = million; ^B^ Total reads mapped to the 2.06 Mb sequence used in the capture array, M = million. The entire chicken genome (*Gallus gallus* v2.1 (galGal3) assembly (WASHUC2, May 2006)) was used in the mapping (chr 1–28, W, and the 995 kb Z) rather than only the three linked chromosomal segments; ^C^ Average fold sequence coverage of the 2.06 Mb targeted region; ^D^ Region sequenced refers to the dp-1, wg-2, or co (596, 466, and 995 kb, respectively) sequence information in which RNA-bait probes were generated from and sequence data aligned to. For example, of the 595 kb dp-1 sequence information for which RNA-bait probes were generated, 96.9% or ~577 kb had sequence reads map to it. Thus 3.1% of the region had no reads mapped to it; ^E^ A gap was defined as any fragment of DNA absent in a genetic line relative to the reference genome sequence. A gap could be due to: (1) an RNA-bait probe was not designed correctly; (2) the NGS technology failed to sequence the fragment of DNA due to sequence structure/quality (e.g., repeat, GC-rich); (3) the reference genome was composed of unknown sequence (N) and therefore a probe could not be generated for that region but the size of the fragment was still known and accounted for in the reference genome; and (4) the genetic line of interest does not have that fragment of DNA, *i.e.*, the gap is a “true deletion”.

**Table 3:** Assessment of SNPs and micro-indels within three congenic lines: Diplopodia-1.003, Wingless-2.331, Coloboma.003.

Chr	# of SNPs in Sequenced Region			# of Short Indels (1–23 nt) in Sequenced (2.06 Mb) Region					
	Dp-1.003	Wg-2.331	Co.003	Dp-1.003		Wg-2.331		Co.003	
				Insertions	Deletions	Insertions	Deletions	Insertions	Deletions
1	*2,593*	2,434	2,478	*150*	*133*	116	130	109	110
12	1,245	*1,724*	1,225	79	101	*108*	*138*	71	93
Z	2,903	1,787	*2,500*	128	171	150	185	*125*	*155*

Total number of SNPs, insertions, and deletions identified within the targeted regions of interest.

**Figure 3:**
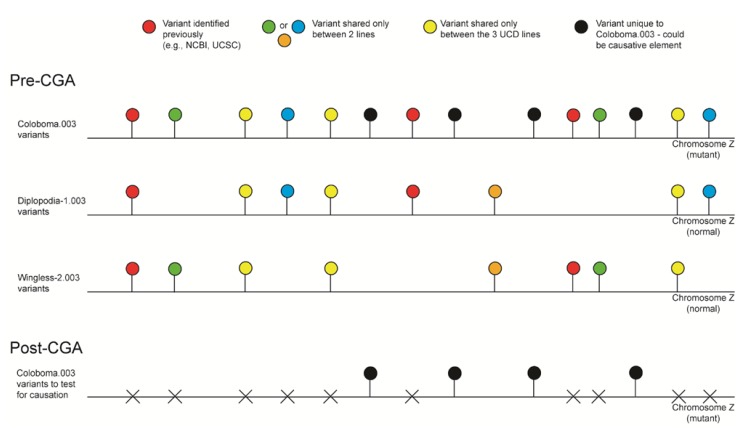
Illustration of the comparative genomic analysis strategy to identify mutant-specific polymorphisms. Comparative genomic analyses (CGA) were conducted to eliminate SNPs and indels shared between two or more of the congenic lines or to previously identified polymorphisms. Those variants not observed in any other genetic line or chicken species were denoted as unique and potential causative elements. An example, to illustrate how this analysis was performed, is shown in reference to the UCD-Coloboma.003 linked region on chromosome Z. All three genetic lines were assessed at the same location for polymorphisms, those variants shared between Co.003 and any other line were removed. Only those elements unique to that region in the Coloboma.003 genetic line are of interest and should be further assessed for causation.

Multiple pairwise-line comparisons (a.k.a. comparative genomic analysis) were conducted to eliminate SNPs and indels shared between two or more of the congenic lines or to previously identified polymorphisms found in databases (see Figure 3). We identified a total of 6104 SNPs, 245 insertions, and 299 deletions shared among the congenic lines within the linked regions of GGA 1, 12, and Z; these results are suggestive of polymorphisms found within the highly inbred UCD-003 genetic line and will be useful in creating future SNP arrays (NCBI accessions: ss472336609-ss472343089). The pairwise-line comparison indicated 2110 novel SNPs, 201 novel insertions, and 219 novel deletions unique to the three genetic lines, *i.e.*, the respective introgressed regions. Assessment of homozygous and heterozygous SNP loci reduced the size of the linked regions by 1.23 Mb in total, with an average of 276 kb remaining as linked to each mutation (Table 1 and Table 2). Further, assessment of the SNPs alone allowed us to eliminate eight genes as causative for the mutant phenotypes, a major advantage for future functional studies. Similarly, micro-indels (1–3 nt) and gaps were identified (Table 2) and reduced in the same fashion by multiple pairwise-line comparison. These variants will be assessed for contribution towards the mutant phenotype. Macro-indels (4–27 nt) were also identified; however, upon review of those positioned within the exons and splice sites of Wg-2.331 only, none of the bioinformatically predicted macro-indels (n = 16) were present (data not shown). The 0% validation rate for the macro-indels indicates a custom script problem; this script will be revised and macro-indel location re-evaluated. Further assessment of the unique sequence elements identified (SNPs, micro-indels, gaps) include, but are not limited to, identification of the position of each element in the genome (e.g., exon, intron, *etc*.) and codon and amino acid modifications caused by the element (Figure 2, step 7). Such knowledge allows prioritization of the elements for further validation (Figure 2, step 8–9).

### 2.3. Reference-Assisted *De Novo* Assembly

Genomic alignment programs are utilized to: (1) identify putative inversions and other genomic rearrangements; (2) confirm the current reference genome assembly; (3) assess gaps in the sequence information (a gap can be present due to sequencing problems or because the gap is a deletion in a particular line); and (4) identify novel genomic sequence. Reference-assisted *de novo* assembly was therefore conducted using the Mauve 2.3.1c software [10] on the 72.7M reads generated for Dp-1.003, Wg-2.331, and ­Co.003 genetic lines (Table 2). Seven independent Mauve alignments were conducted to identify contig-alignment program bias. This included four three-chromosome alignments (Supplemental Figure 1), utilizing only the linked regions of GGA 1, 12, and Z, and three individual chromosome assemblies (Supplemental Figure 2). From these reads Mauve generated an average of 81 contigs ≥400 bp for each of the three-chromosome assemblies; 89.4% of these contigs showed high sequence similarity (≥90%) to the reference genome. The program results indicated several translocation events. Validation of the chromosomal rearrangements and identified gaps using a variety of techniques (e.g., FISH, re-sequencing) is necessary in order to assess legitimacy of any predicted chromosomal rearrangement. 

## 3. Experimental Section

### 3.1. Genetic Lines

We investigated three developmental mutations bred into congenic lines: Diplopodia-1 (Dp-1).003, Wingless-2 (Wg-2).331, and Coloboma (Co).003. Two of the single-gene mutations (*dp-1*, *co*) were backcrossed onto the highly inbred (F >0.99) Single Comb White Leghorn (SCWL) UCD-003 and the third (*wg-2*) was bred to be congenic on UCD-331, which is a congenic line on the UCD-003 background except for the major histocompatibility complex [1,23]. The congenic line series provides a unique advantage for employing capture array technology and subsequent analyses as each line serves as a control genotype for the other two since the mutants map to three different chromosomal regions. Phenotypes, SNP-genotyping, and mapping of the mutant chromosomes and CRs are described by Robb *et al*. [1]. Animals used for the study were under the care and supervision of trained staff and as per protocols approved by the UC Davis Institutional Animal Care and Use Committee.

### 3.2. Sample Collection, Capture Array Sample Preparation, and SOLiD ™ Sequencing

Embryos were incubated to E10, an age of development when the phenotypes are easily discerned [normal +/+ and +/−; mutant −/− (autosomal) or −/W (sex-linked)]. DNA was obtained from three tissues (brain, heart, and liver) and purified using the DNeasy^®^ Blood & Tissue kit (Qiagen). Mutant status was also confirmed by genotyping using primers linked to each mutation [1].

As *co* is sex-linked, we used females which increased the probability that the mutant Z region would be sequenced (in birds, the female is the heterogametic sex (ZW) while the male is the homogametic sex (ZZ)). Thus, male (ZZ)/female (ZW) status was determined based on phenotype (gonadal examination) and genotype. The latter employed standard PCR of two sex-chromosome-specific loci [1]. Reactions were amplified using Phire^®^ Hot Start II DNA Polymerase (Finnzymes) and amplicons sized by electrophoresis (1.5% gel, 1× TAE, 100 V, 2 h). Primers were GGA_W_F (5′ -CTGACTACCTTTGCAGTGCT- 3′), GGA_W_R (5′ -GCTGAGAAACTTATCCCTCA- 3′), GGA_Z_F (5′ -AAAGCAAAGGTTTTTGTTCC- 3′), and GGA_Z_R (5′ -TGGAAATGCCTGCTAAACTA- 3′). Amplicon sizes were 227 bp (GGA W) and 161 bp (GGA Z).

Line-specific DNA pools (50 μg/line of two female (25 μg each) mutant samples/line), were sent to SeqWright DNA Technology Services (Houston, TX) for target enrichment and SOLiD ™ V3 Plus Platform sequencing (Applied Biosystems, Foster City, CA). Only 3 μg of DNA is needed to generate a library so the amount sent was in large excess. SeqWright’s Genomic Enrichment Services utilized Agilent’s (Santa Clara, CA) SureSelect Target Enrichment System in order to enrich for three specific regions on chromosomes 1 (Dp-1.003), 12 (Wg-2.331) and Z (Co.003) using overlapping RNA sequence “baits” (120-bp in length, complementary to the targeted region) selected specifically to provide complete coverage for each genomic region of interest [1]. GGA 1 and 12 reference sequences for probe design were obtained from NCBI [24] and GGA Z sequence information from Drs. D. Winston Bellott and David Page (Whitehead Inst., MIT) [9]. Preceding target enrichment and sequencing, a fragment library (base pair peak of 150–200) was constructed for each genetic line. Each library was then tagged with a different barcode (a.k.a. indexing-specific adapter) so that the individual genetic lines were distinguishable; the three libraries were pooled prior to emulsion PCR and SOLiD™ sequencing. See Figure 1 and Figure 2 for more detail as to the CA/NGS methodology.

### 3.3. Sequence Assembly

Colorspace read data (.csfasta, .stats, .qual) were sorted by the barcodes using custom Python scripts; barcodes were removed and reads (50 bp length) were trimmed by three nucleotides on each end. The reads were then mapped to the chicken reference genome (*Gallus gallus* v2.1 (galGal3) assembly (WASHUC2, May 2006) [25]) using Bowtie 0.12.5 [26], allowing for ≤2 mismatches within each read for initial assessment of alignment quality. All alignments were converted to Sequence Alignment/Map (.sam) format and were viewed using Samtools 0.1.7a [27]. Bioinformatic raw data and mapped alignments were outsourced to the University of California, Davis (UCD) Bioinformatics Core [28] for variant identification and reference-assisted *de novo* assembly. 

### 3.4. Reference-Assisted *De Novo* Assembly

Mauve 2.3.1c, a multiple genome alignment software [10], was utilized to identify potential chromosomal rearrangements; these events were identified by analyzing the position of the Locally Collinear Blocks (LCBs) within each genetic line relative to each other. The program first utilizes the original, sorted sequence reads from the three mutant genomes to generate contigs *de novo*. Upon completion, each contig is aligned to the reference genome, thereby identifying its position in the genome. Herein this alignment is referred to as the reference-assisted *de novo* assembly.

### 3.5. SNP, Micro- and Macro-Indel, and Sequence Gap Discovery

The trimmed read data and alignment files were provided to the UC Davis Bioinformatics Core wherein read format conversion and alignment using BWA [29,30], SAMtools [27,31], and custom Perl scripts were run. Output files included information on SNPs, micro-indels (1–3 nt) and macro-indels (4–27 nt) for all three mutant regions. Filters for the identification of SNPs and micro-indels include: read coverage ≥10, root-mean-square (RMS) mapping quality of the aligned reads ≥30, and the minimum variant frequency/count ≥20% (*i.e.*, ≥2 mutant read variants per 10 reads). Note that SNP and micro-indel identification was carried out for each genetic line individually, as reads were sorted by barcode prior to alignment and variant discovery. Additionally, gaps (unsequenced segments/coverage gaps) within the 2.06 Mb targeted region were identified using BEDTools [32,33]. Those genomic gaps only identified in one line (but present in the others) were considered putative deletions and should be confirmed for legitimacy.

To evaluate the accuracy of variant calling, SNPs identified as linked to each mutation using the 3K and 60K SNP arrays [1] were identified as present in the CA results. Additionally, both SNPs and micro-indels (1–3 nt) were compared to previously identified variants found in other chickens (*i.e.*, Silkie, commercial egg- and meat-type birds assessed by the Beijing Genomics Institute, [34]) [NCBI, UCSC Genome Browser].

### 3.6. Capture Enrichment Data Analyses

Sequences were initially identified as polymorphic relative to the *Gallus gallus* NCBI reference genome sequence, UCD-001 (Red Jungle Fowl (RJF)) [2]. However, since such differences would include normal variation (unrelated to the mutation due to line divergence between the RJF and SCWL), we reduced this variation by employing a comparative genomic analysis between and among the three mutant lines (Figure 3). Previously, we had shown that the UCD developmental mutant lines varied at only the causative region (CR) while the remaining genome was largely identical to UCD-003 [1]; consequently, each mutant line serves as a control/reference sequence for the other two mutations since the mutations are linked to different chromosomes.

Thus, our strategy for mutant-specific sequence discovery was the following: after sequence variant identification and genome comparisons, any polymorphism identified as unique, defined as present only in the mutant line of interest and not found in the other two lines, the reference genome, or any previously identified polymorphism reported in NCBI and the UCSC Genome Browser, was considered a candidate for being the causative element for the respective developmental mutation (Figure 3). 

## 4. Conclusions

With the price for NGS technologies decreasing at exponential rates, this state-of-the-art technology is now a realistic research tool for many biological laboratories desiring genomic sequence information to complement biological analysis of phenotypes. However, two limiting factors still exist, thereby discouraging the use of NGS in smaller labs. Such deterrents include the lack of bioinformatics expertise and a “draft-stage” reference genome. To overcome these two issues we: (1) outsourced our sequencing data to an affordable, local bioinformatics core and received results within a reasonable period of time and (2) utilized not only the closely related species reference genome (*Gallus gallus versus G. domesticus*) but also congenic line partners for alignment, variant discovery and elimination. Beyond contributing to our analysis of the mutations, the information generated contributes to an improved understanding of the reference genome, inclusive of the filling-in of sequence gaps and basic information of sequence content of particular chromosomes (e.g., autosomal *vs.* sex) (unpublished data).

In summary, the utilization of the CA/NGS technology resulted in the narrowing of the causative region size for each of the three mutations studied. Further, our multiple pairwise-line comparison strategy eliminated those shared polymorphisms unrelated to the syndromes. Sequencing verification of the identified SNP, insertions, and deletions using additional mutant samples will indicate if a particular polymorphism remains linked, *i.e.*, a validation step [11]. Ultimately, to establish the role of a sequence element in causing the mutant phenotype, such sequence variants will be analyzed using the appropriate functional assays to determine cause/effect.

## Supplementary Files

Supplementary File 1 PDF-Document (PDF, 661 KB)
