# Supplementary material for: Case Study of Sequence Capture Enrichment Technology: Identification of Variation Underpinning Developmental Syndromes in an Amniote Model
Source: Genes (Basel). 2012 Mar 26;3(2):233–47. doi: 10.3390/genes3020233 (PMC3899949; doi:10.3390/genes3020233)
Supplement: Supplementary File 1 — PDF-Document (PDF, 661 KB) [file genes-03-00233-s001.pdf]

Supplemental Figure 1. Three-chromosome (1, 12, Z) region Mauve alignments: Combinatorial comparisons among three developmental mutant congenic lines and the reference genome

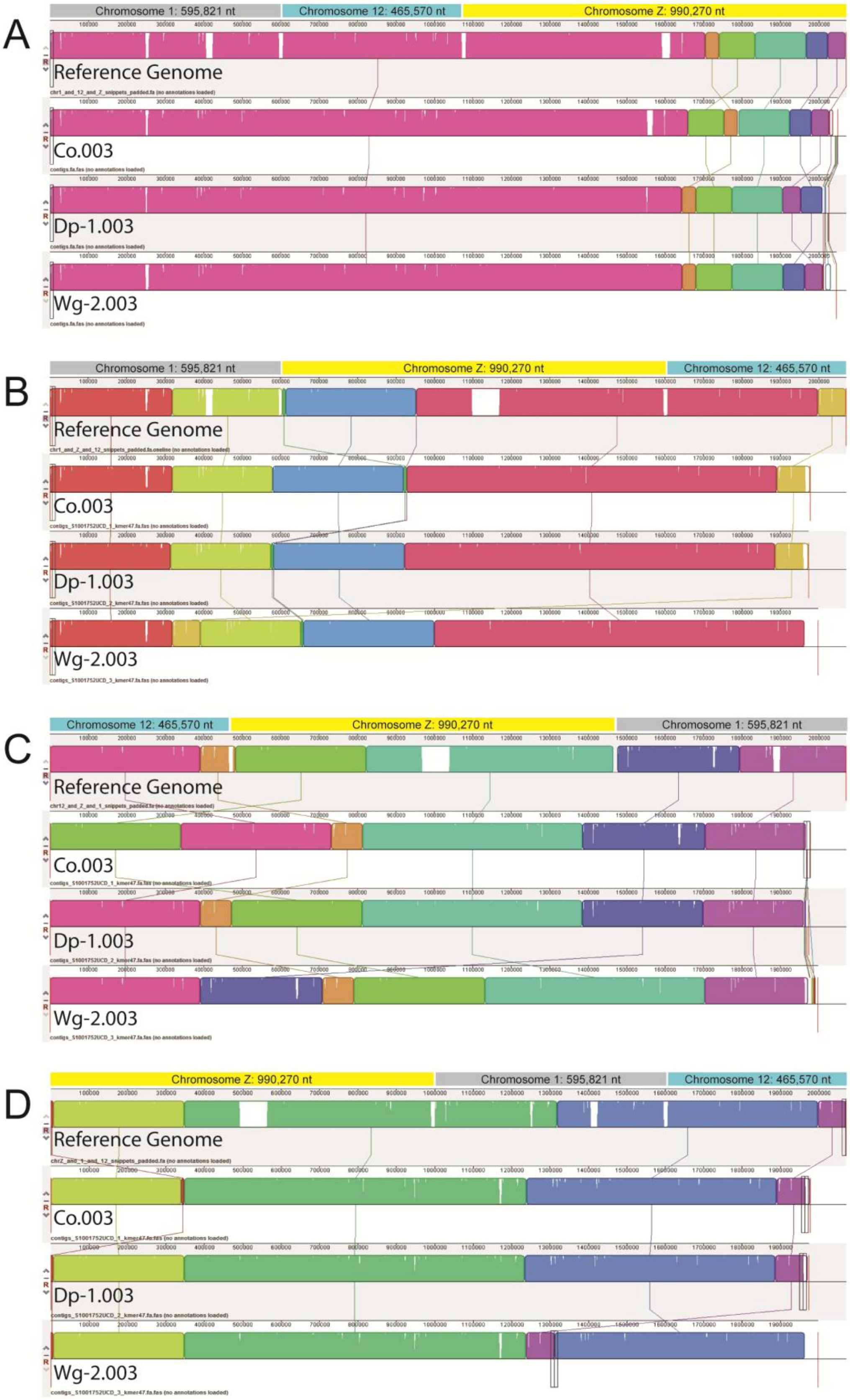

Four sets of combinatorial three-way-chromosome alignments were generated in order to identify chromosomal rearrangements specific to each genetic line. We carried out reference-assisted *de novo* assembly using the Mauve software 2.3.1c [10] on the 72.7M reads generated for *dp-1*, *wg-2*, and *co* genetic lines (Table 2), see Methods. As shown, a total of four, three-chromosome alignments were generated. Alignments utilized only the linked regions of chr 1 (595,821 nt, indicated with a gray bar across the top of the alignment), 12 (465,570 nt, indicated with a teal bar across the top of the alignment) and Z (994,523 nt, indicated with a yellow bar across the top of the alignment). Several alignments, changing the order of the chromosomes, were conducted to identify if a particular chromosomal aberration was generated due to software alignment bias. Information from these assemblies allowed us to: (1) identify putative chromosomal rearrangements among the three congenic lines; (2) confirm the current reference genome assembly; (3) served as a method of assessing gaps in the sequence information (i.e., is a gap present due to sequencing issues or because the gap is actually a genomic deletion in a particular line); and (4) to identify novel genomic sequence. Several putative translocation events were identified among the various chromosomal orientations, these require further study and verification.

A. The three-chromosome alignment order is chr 1-12-Z. A translocation event (at positions: ~1,700,000 and ~1,800,000) in the Co.003 genetic line on chromosome Z (the mutant chromosome) relative to the other genetic lines (reference genome, Dp-1.003 and Wg-2.331) is indicated (note: orange and lime green blocks). A similar translocation event can be seen in alignments 1B (green/blue blocks) and 1D (red/lime green blocks) as well as Supplemental Figures 2A-C. Additionally, using this alignment, a translocation event (1,900,000 and 1,950,000) can be seen in Dp-1.003 (purple and blue blocks) on chromosome Z while the other two developmental mutant congenic lines display the reference genome orientation.

B. The three-chromosome alignment order is chr 1-Z-12. A translocation event (green and blue blocks; positions: ~600,000 and ~900,000) is observed in the Co.003 genetic line on chromosome Z (the mutant chromosome) relative to the other genetic lines (reference genome, Dp-1.003 and Wg-2.331). A similar alignment was predicted in Supplemental Figure 1A and 1D as well as Supplemental Figures 2A-C. Additionally, this alignment shows a chromosome translocation (yellow block) in the Wg-2.331 genetic line (positions: ~350,000 and ~1,900,000) in which a portion of chromosome 12 has been aligned to chromosome 1. This particular translocation event can be seen in alignment 1D (purple/blue blocks).

C. The three-chromosome alignment order is chr 12-Z-1. A translocation event (green block; positions: ~1 and ~500,000) can be seen in the Co.003 genetic line on chromosome Z (the mutant chromosome) and 12 (non-mutant) was observed. The reference genome, Dp-1.003 and Wg-2.331 do not have this chromosomal rearrangement. Additionally, a translocation event (purple block; positions: ~400,000 and ~1,400,000) is observed in the Wg-2.331 genetic line wherein a segment from chromosome 1 has been aligned to chromosome 12.

D. The three-chromosome alignment order is chr Z-1-12. A translocation event (red and lime green blocks; positions: ~1 and ~350,000) in the Co.003 genetic line on chromosome Z (the mutant chromosome) is observed relative to the other genetic lines (reference genome, Dp-1.003 and Wg-2.331). A similar translocation event can be seen in alignment 1A (orange/lime green blocks) and 1B (green/blue blocks) as well as Supplemental Figures 4A-C. Wg-2.331 also displays a translocation event (positions: ~1,200,000 and ~1,900,000) wherein a portion of chromosome 12 (purple block) has been localized to chromosome 1 (as also seen in alignment 1B).

Supplemental Figure 2. Individual-chromosome Mauve alignments: Combinatorial comparisons among three developmental mutant congenic lines and the reference genome

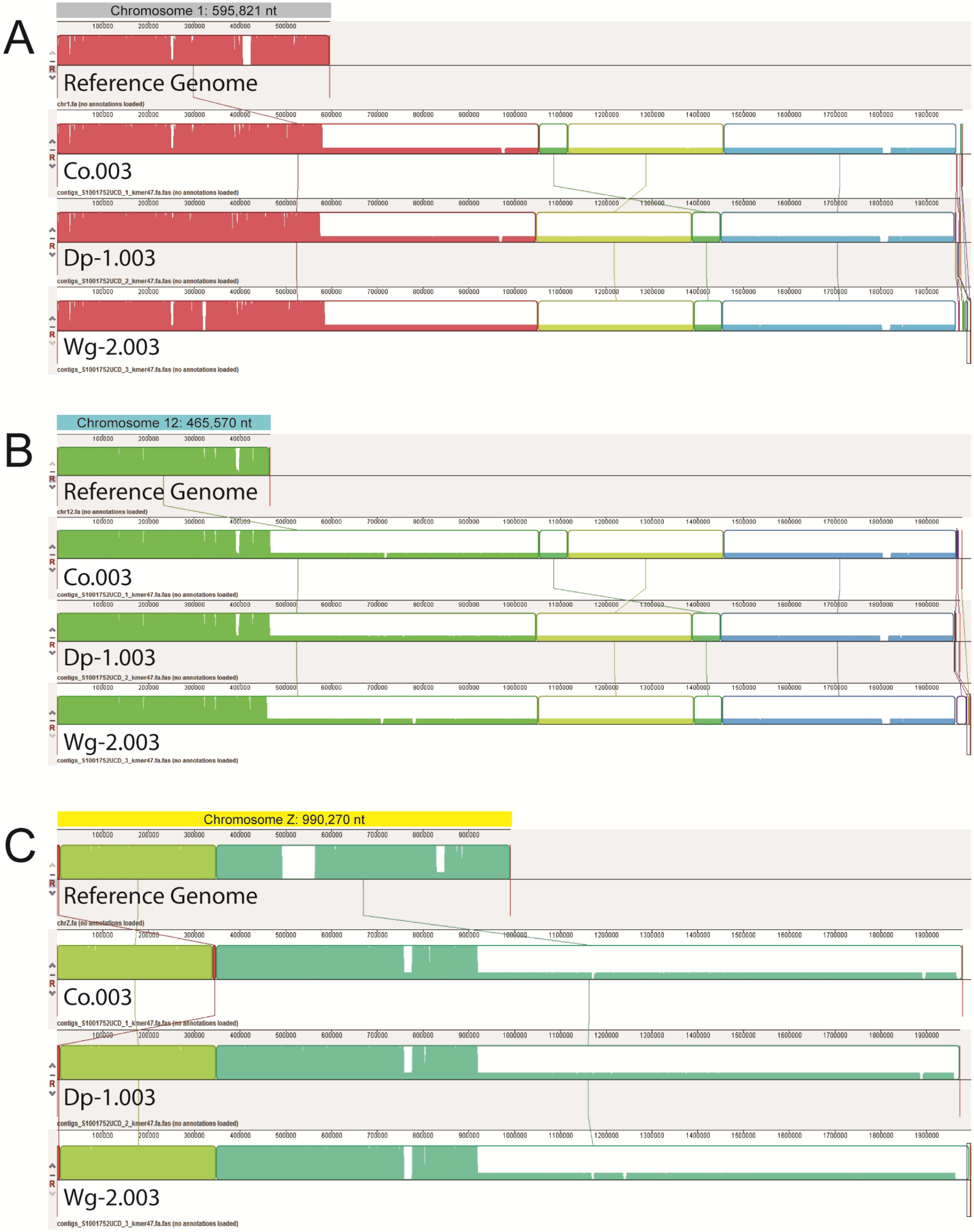

Three sets of single-chromosome alignments were generated in order to identify chromosomal rearrangements specific to each genetic line. We carried out reference-assisted *de novo* assembly using the Mauve software 2.3.1c [10] on the 72.7M reads generate d for *dp-1*, *wg-2*, and *co* genetic lines (Table 2), see Methods. Alignments utilized only a specific chromosomal region (i.e., chromosome 1, 12 or Z) rather than three contiguous alignments (shown in Supplemental Figure 1). Chromosomal regions are indicated as follows: chr 1 - 595,821 nt, indicated with a gray bar across the top of the alignment; chr 12 - 465,570 nt, indicated with a teal bar across the top of the alignment; and Z - 994,523 nt, indicated with a yellow bar across the top of the alignment. Information from these assemblies allowed us to: (1) identify putative chromosomal rearrangements among the three congenic lines; (2) confirm the current reference genome assembly; (3) served as a method of assessing gaps in the sequence information (i.e., is a gap present due to sequencing issues or because the gap is actually a genomic deletion in a particular line); and (4) to identify novel genomic sequence. Overall, utilizing various chromosomal orientations, we have identified several putative translocation events in all three genetic lines.

A. Single-chromosome alignment for chr 1 only. No chromosomal aberrations indicated. However, since the reads for each developmental genetic line included those from all three genomic regions, contigs were still produced and aligned to the right of chr 1 (see white blocks). A translocation event (green and yellow blocks at positions ~1,100,000 (white-green) and ~1,400,000 (white-yellow)) in the Co.003 genetic line on chromosome Z (the mutant chromosome) remains indicated relative to the other two congenic lines (Dp-1.003 and Wg-2.331). This translocation event can also be seen in alignment Supplemental Figure 2B whereas in 2C a Z chromosomal translocation is also indicated, but smaller (red/green blocks).

B. Single-chromosome alignment for chr 12 only. No chromosomal aberrations were found on 12. However, a translocation event (white-green and white-yellow blocks at positions ~1,100,000 and ~1,400,000) in the Co.003 genetic line on chromosome Z (the mutant chromosome), relative to the other two congenic lines (Dp-1.003 and Wg-2.331), can be seen to the right of the chr 12 alignment. This translocation event can also be seen in alignment Supplemental Figure 2A whereas in 2C a Z chromosomal translocation is also indicated, but smaller (red/green blocks).

C. Single-chromosome alignment for chr Z only. A translocation event (red and green blocks at positions ~1 and ~350,000) can be seen on chromosome Z within the Co.003 genetic line relative to the other genetic lines (reference genome, Dp-1.003 and Wg-2.331). Similar translocation events can also be seen in alignment 2A and 2B as well as in the three-chromosomal alignments displayed in Supplemental Figure 1 A, B, D. Additionally, reference genome sequence information found between position ~500,000 and ~550,000 (white block) did not have any sequence information aligned to it from the three developmental mutant genetic lines.
